# Stepping stability: effects of sensory perturbation

**DOI:** 10.1186/1743-0003-2-9

**Published:** 2005-05-27

**Authors:** Chris A McGibbon, David E Krebs, Robert Wagenaar

**Affiliations:** 1 Institute of Biomedical Engineering, University of New Brunswick, 25 Dineen Drive, Fredericton, New Brunswick E3B 5A3, Canada; 2 Massachusetts General Hospital, Biomotion Laboratory, Boston, MA 02114, USA; 3 MGH Institute of Health Professions, Boston, MA 02114, USA; 4 Department of Physical Therapy, Sargent College of Health and Rehabilitation Sciences, Boston University, Boston, MA 02114, USA

**Keywords:** stability, auditory perturbation, stepping, locomotion, vestibular, cerebellar

## Abstract

**Background:**

Few tools exist for quantifying locomotor stability in balance impaired populations. The objective of this study was to develop and evaluate a technique for quantifying stability of stepping in healthy people and people with peripheral (vestibular hypofunction, VH) and central (cerebellar pathology, CB) balance dysfunction by means a sensory (auditory) perturbation test.

**Methods:**

Balance impaired and healthy subjects performed a repeated bench stepping task. The perturbation was applied by suddenly changing the cadence of the metronome (100 beat/min to 80 beat/min) at a predetermined time (but unpredictable by the subject) during the trial. Perturbation response was quantified by computing the Euclidian distance, expressed as a fractional error, between the anterior-posterior center of gravity attractor trajectory before and after the perturbation was applied. The error immediately after the perturbation (Emax), error after recovery (Emin) and the recovery response (Edif) were documented for each participant, and groups were compared with ANOVA.

**Results:**

Both balance impaired groups exhibited significantly higher Emax (*p *= .019) and Emin (*p *= .028) fractional errors compared to the healthy (HE) subjects, but there were no significant differences between CB and VH groups. Although response recovery was slower for CB and VH groups compared to the HE group, the difference was not significant (*p *= .051).

**Conclusion:**

The findings suggest that individuals with balance impairment have reduced ability to stabilize locomotor patterns following perturbation, revealing the fragility of their impairment adaptations and compensations. These data suggest that auditory perturbations applied during a challenging stepping task may be useful for measuring rehabilitation outcomes.

## Introduction

Balance and postural control in humans is often studied by measuring the sway and/or muscle EMG response to a controlled mechanical perturbation, mainly taking the form of forward and backward or side-to-side platform translations, and foot dorsi- and plantar-flexing rotations [1-7]. Perturbations have also taken the form of a sudden push or pull to the upper body or waist while subjects stand or walk [8-13]. While these studies provide a better understanding of postural reflexes to mechanical perturbations, the conditions for the responses often do not correspond to the natural conditions in which individuals with balance impairments fall. Falls in individuals with balance impairments mainly occur during common, everyday activities [14-16]. Individuals with balance impairments are also susceptible to self-initiated perturbations (cognitively or externally cued but without external forces) such as sudden stops [17,18], turns [19], or stepping corrections to avoid obstacles [20,21].

Numerous studies on balance and postural control from the perspective of non-linear dynamics have been published in the last decade [22-28]. Collins et al. [22] applied the analysis of Brownian motion (stabilogram-diffusion analysis) to undisturbed standing and concluded that, compared to young healthy subjects, elderly subjects utilized open-loop control schemes for longer periods of time before closed-loop feedback mechanisms were initiated, but that their closed-loop postural control mechanisms were more stable. Mitchell et al. [25] used stabilogram-diffusion analysis to show that people with Parkinson's disease (PD) compensate for less stable open-loop control in the anteroposterior direction with increased closed-loop control in mediolateral direction. Van Emmerik et al. [23] applied dimensionality analysis to quiet standing of healthy people and people with tardive dyskinesia, and reported that loss of variability, rather than high sway amplitude, may cause postural instability.

Studying the relative phase dynamics between the movements of upper and lower extremities as a function of walking velocity in healthy persons and people with PD, van Emmerik and Wagenaar [29] reported that in PD persons the ability to switch between coordination patterns (flexibility) was reduced whereas the within-pattern variability was decreased (hyperstability) compared to healthy participants. This finding was consistent with the neurological symptom 'rigidity' assessed by means of the Columbia rating scale. Results were also corroborated by van Emmerik et al. [30], who reported smaller changes in mean relative phase between transversal pelvic and thoracic rotations and a lower variability in relative phase in a PD group compared to a group of healthy individuals. The locomotor stability of people with other neurologic deficits, such as vestibular hypofunction and cerebellar pathology, has received less attention [31-34], and has not been assessed during perturbed locomotor tasks.

The objective of the present study was to investigate the stability of stepping in people with peripheral and central vestibular dysfunction by means of an easily controlled sensory (auditory) perturbation test that is functional and self-initiated (via external cue). We have previously reported a cadence controlled, repeated bench stepping task for studying people with vestibular [33,34] and cerebellar pathology [32]; our results show this activity challenges participants' locomotor and balance systems. In this report, we applied an auditory perturbation by suddenly changing the cadence of the metronome (100 beat/min to 80 beat/min) at a predetermined time during the trial. The effects of the perturbation on the stability of the movement patterns were studied by applying tools derived from non-linear dynamics. We hypothesized that, when compared to healthy participants, 1) balance impaired participants (vestibular hypofunction and cerebellar pathology) would demonstrate more variability when the perturbation is applied, and 2) recover more slowly from the perturbation. This study should be useful in the development of new approaches for assessing treatment efficacy.

## Methods

### Participants and Procedures

Participants consisted of five healthy adults (HE: mean age = 43.4 ± 15.5 years), six adults with vestibular hypofunction (VH: mean age = 45.3 ± 10.2 years), and three adults with cerebellar pathology (CB: mean age = 55.6 ± 12.0 years). Sample characteristics are summarized in Table 1. HE participants were free of orthopaedic, neurologic or other conditions affecting physical performance or balance. Participants with CB were diagnosed by a neurologist's examination of the patients' signs and symptoms and from Magnetic Resonance or Computed Tomography brain scans [35]. Participants with VH were diagnosed using a vestibular test battery and by an otoneurologist's examination as either bilaterally (BV) or unilaterally (UV) deficient [36,37]. BV was diagnosed as abnormal vestibulo-ocular reflex gains (at least 2.5 standard deviations below normal) on computerized sinusoidal vertical axis rotation testing, and bilaterally absent caloric responses as determined by cold and warm water stimulation. UV was diagnosed by demonstration of at least one of the following: 30% unilaterally reduced caloric response, positional nystagmus while lying with the damaged ear down, and confirmatory abnormalities on rotational testing. Beyond their respective primary diagnoses, persons with VH and CB had no evidence of other conditions that could affect balance control. All participants signed informed consent forms prior to testing according to institutional guidelines on human research. Specific diagnoses are listed for each participant in Table 2.

**Table 1:** Subject characteristics

	Age (yrs)	Height (m)	Weight (kg)*
Healthy Participants (5 females)			
Mean	43.4	1.58	53.6
St. Dev.	15.5	.18	5.0
Range	24.2 – 59.58	1.22 – 1.73	45.0 – 59.1
Vestibular Hypofunction Participants (5 females, 1 male)			
Mean	45.3	1.67	92.6
St. Dev.	10.2	.09	28.7
Range	29.92 – 61.60	1.55 – 1.83	54.55 – 145.45
Cerebellar Pathology Participants (2 females, 1 male)			
Mean	55.61	1.63	73.87
St. Dev.	11.99	.08	15.09
Range	39.58 – 68.42	1.55 – 1.73	56.36 – 93.18

* Significant between-groups difference for healthy vs. vestibular hypofunction participants (*p *= .05)

**Table 2:** Individual subject diagnoses and perturbation error responses.

Participant	Diagnosis	*Emax	^†^Emin	^‡^Edif
1	HE – Healthy	.26	.10	62.0
2	HE – Healthy	.31	.15	52.6
3	HE – Healthy	.39	.15	60.5
4	HE – Healthy	.42	.13	68.6
5	HE – Healthy	.46	.17	63.7

6	VH – Unilateral vestibular hypofunction	.46	.11	77.0
7	VH – Unilateral vestibular hypofunction	.56	.23	59.2
8	VH – Bilateral vestibular hypofunction	.61	.34	44.9
9	VH – Unilateral vestibular hypofunction	.61	.16	73.6
10	VH – Unilateral vestibular hypofunction	.91	.23	74.3
11	VH – Unilateral vestibular hypofunction	.94	.27	71.1

12	CB – Idiopathic spinocerebellar degeneration	.52	.26	49.3
13	CB – Cerebellar dysfunction	.64	.22	65.3
14	CB – Idiopathic spinocerebellar degeneration	.94	.39	59.0

*Emax = Maximum fractional error at initiation of perturbation; ^†^Emin = Fractional error recovery at 2–3 cycles after perturbation; ^‡^Edif = Percent difference in fractional error response from initiation to recovery.

**Figure 1:**
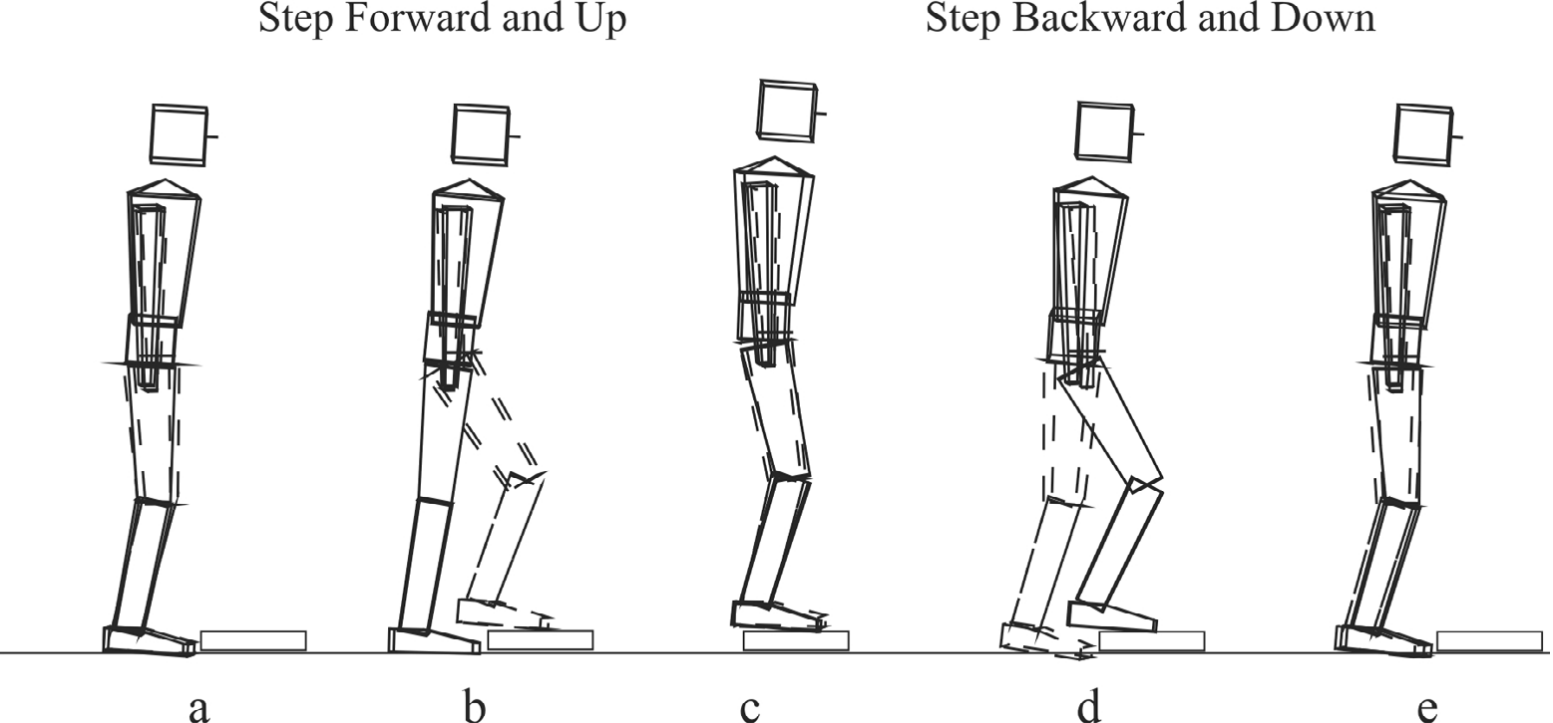
Three-dimensional android reconstruction of a representative healthy subject performing the stepping task. (a-b-c) The subject steps forward onto the platform with their dominant leg; (c-d-e) steps backward off the platform with their dominant leg. The task is performed repeatedly over a 30 second period (approximately 12 cycles).

Participants performed 30 second repeated bench stepping trials using a step up forward/step down backward paradigm: participants were instructed to step forward onto the platform and then step backward off the platform (Figure 1), leading with their dominant leg, and synchronizing their foot strikes with the beats of an electronic metronome. The dominant leg was determined by asking participants to pantomime kicking a ball. The platform consisted of two side-by-side 7.6 × 57.6 × 23.0 cm (height × width × depth) blocks placed on the front halves of two 60 cm long Kistler force plates (Kistler Instruments, Inc. Winterthur, Switzerland). Bilateral, three-dimensional body segment kinematics were collected at 152 Hz with four SELSPOT (Selective Electronics, Inc. Partille, Sweden) optoelectric cameras. The cameras were used to track arrays of infrared light emitting diodes embedded in rigid plastic disks, securely strapped to eleven body segments (both feet, shanks, thighs and upper arms, and pelvis, thorax and head). Whole body center of gravity (CG) was computed as previously described by Riley et al. [38] Briefly, center of mass in the global reference frame of each of the eleven body segments during a trial were multiplied by their corresponding segment masses, summed, and divided by the total body mass, to arrive at the whole body CG position as a function of time.

Participants performed one-to-two unperturbed stepping trials (constant cadence), followed by one cadence perturbation stepping trial. Perturbation trials were performed by changing (within one beat) the metronome frequency during the stepping trial from 100 to 80 beats per min (bpm) at 10 seconds into the trial, and then from 80 to 100 bpm at 20 seconds into the trial. There were two exceptions: one healthy subject continued at 80 bpm instead of returning to 100 bpm at 20 seconds, and one cerebellar pathology patient, who was unable to reach 100 bpm cadence, performed the trial at 80-60-80 bpm. Participants were aware that the cadence would change during the perturbation trial, but not when it would change.

### Data Analysis

A two-dimensional phase plot was constructed from the anterior/posterior (A/P) velocity component of the whole body CG, *X*(*t*) versus *X*(*t+T*), where *X *was the order parameter (in this case A/P velocity of the CG), *t *was time, and *T *the lag time. The appropriate lag time was determined from the first inflection point (zero crossing) of the autocorrelation function of *X*(*t*). To simplify the analysis description, we use *x*(*t*) = *X*(*t*) and *y*(*t+T*) = *X*(*t+T*).

To represent the perturbation response, the attractor trajectory *x*(*t*), *y*(*t+T*) was compared at each time frame to a reference trajectory *x*_*p*_(*τ*'), *y*_*p*_(*τ*') derived from the attractor trajectory prior to cadence perturbation for each subject. The reference trajectory was generated by first estimating the geometric center *x*_*o*_, *y*_*o *_of the entire attractor time history *t*_*t*_, where *t*_*t *_= 30-T.





A phase angle *φ*(*t*) was then computed from *t *= 0 to *t*_*p *_seconds (at time step 1 / *f *= 1 / 152 Hz = 0.0067 seconds) between *x*(*t*), *y*(*t+T*) and *x*_*o*_, *y*_*o *_from the expression



and forced to range between 0 and 2*π *radians (instead of -*π *to *π*) and converted to degrees. Time *t*_*p *_was 10-T seconds, just prior to onset of the perturbation. The *φ*(*t*) array was then sorted into *φ*'(*τ*), where *τ *was an index array corresponding to ascending values of *φ*(*t*) (from 0 to 360). Attractor dimensions were then sorted into *x*'(*τ*) and *y*'(*τ*) and an *n*th order Fourier series fit was conducted for *x*'(*τ*) and *y*'(*τ*) variables separately, using *φ*'(*τ*) as the independent variable. A 10^th ^order fit was found to minimize the residuals. A new independent variable *φ*_*p*_(*τ*') = 0, 1, 2, ..., 360 was then prescribed and used to compute the reference trajectory coordinates *x*_*p*_(*τ*') and *y*_*p*_(*τ*'), where



The Fourier coefficients were computed from







where *n *= *f*·*t*_*p*_, *f *is the sampling frequency and *t*_*p *_the time duration, *d *is a degree to radian conversion (*π*/180), and *k *is the harmonic index. Computation of *y*_*p*_(*τ*') proceeded in a similar manner.

**Figure 2:**
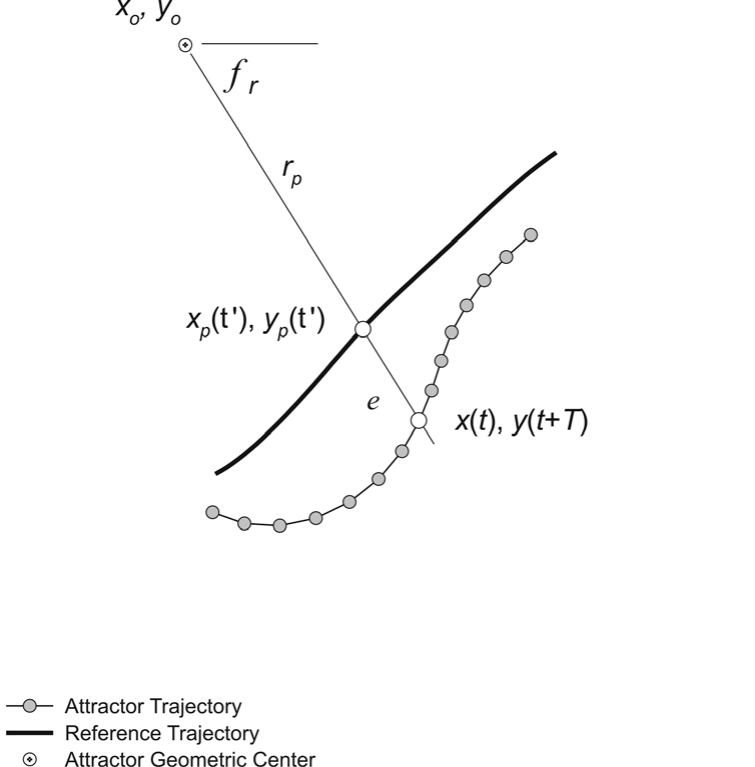
Schematic computation of the attractor trajectory error. All attractor points from time = 0 to 30-*T *seconds are compared to the reference trajectory established for the first 10-*T *seconds based on the squared fractional difference, *ε*, in their radial dimensions from the geometric center of the attractor trajectory orbit.

The perturbation magnitude was estimated by computing the Euclidian distance, expressed as the squared fractional error, *ε*, between the length, *r*, of a line between *x*(*t*), *y*(*t+T*) and *x*_*o*_, *y*_*o *_and length, *r*_*p*_, of a line between *x*_*p*_(*τ*'), *y*_*p*_(*τ*') and *x*_*o*_, *y*_*o*_. The latter dimension was determined by first calculating the angle of *r *(ie. using equation 3), *φ*_*r*_, rounding it to the nearest degree, and using it as an index, *τ*' = *φ*_*r *_to find the corresponding *x*_*p*_(*τ*'), *y*_*p*_(*τ*') coordinates. The error was then calculated from



where *t *= 0 to 30-*T *seconds (see also Figure 2).

To compare groups of participants, the error data for each subject was first binned into 2 second intervals (a total of 5 intervals) between the 10 second and 20 second marks. The peak error was then documented for each bin. The maximum value of the five peaks (Emax, occurring in the first or second bin) and minimum value of the five peaks (Emin, occurring in the last bin) were then recorded for each subject. The magnitudes of Emax and Emin both represent the stability of the participants following the auditory perturbation. The magnitude of Emin also indicates participants' ability to recover. We also analyzed the difference between Emax and Emin (Edif), as a measure of participants' recovery response, relative to their initial perturbation response.

Analysis of variance (ANOVA) was used to compare dependent variables (Emax, Emin and Edif) among groups of participants at an alpha level of .05. All statistical comparisons were conducted using SPSS (v10, SPSS, Chicago, IL).

## Results

There were no significant differences in age (*p *= .50) and height (*p *= .59) between groups, but weight was significantly greater for the VH participants compared to the HE participants only (*p *= .05).

### Cadence Perturbation Analysis

**Figure 3:**
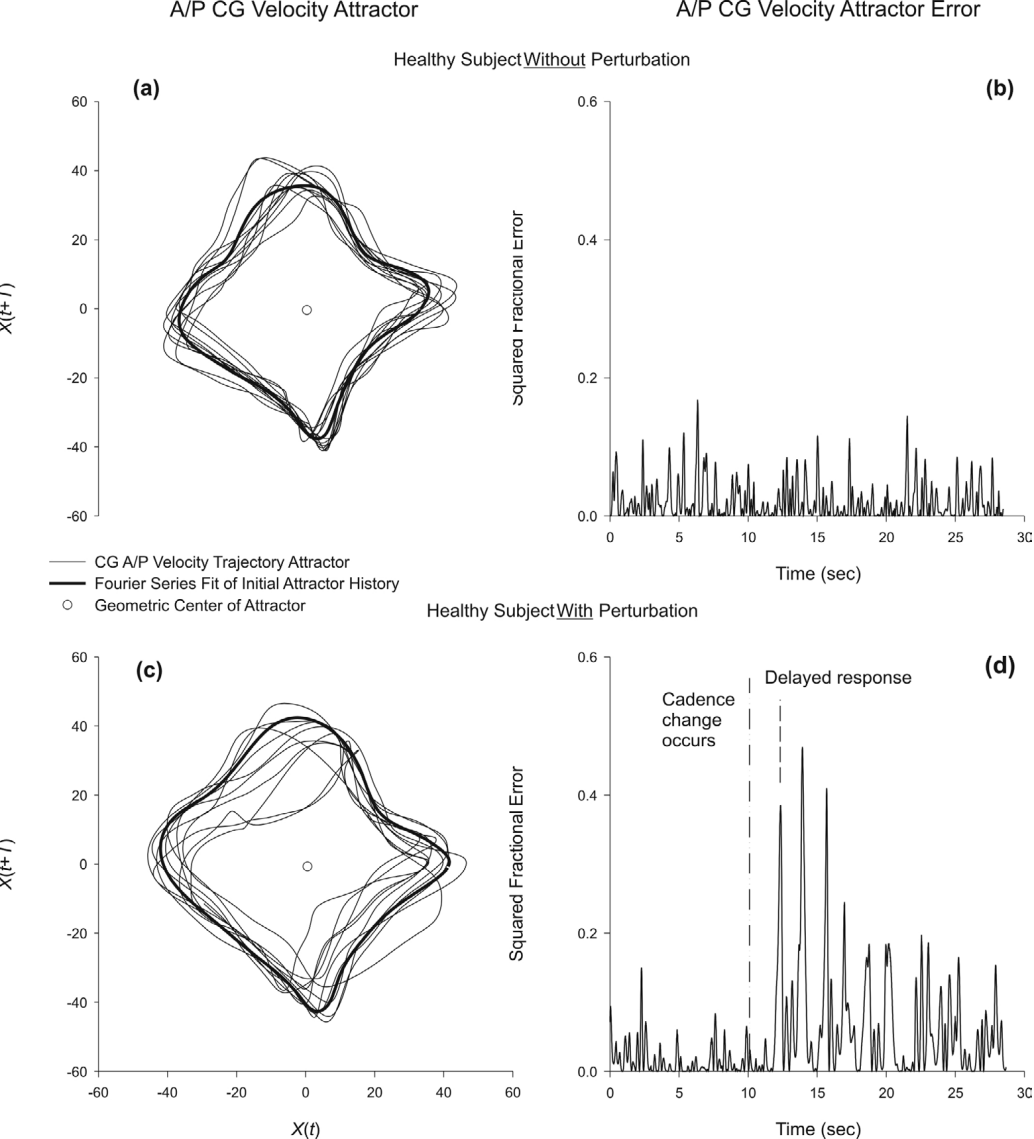
Attractor trajectory error for a representative healthy subject during the stepping task. The top panels are: (a) The A/P CG velocity attractor during an unperturbed cadence trial; (b) The attractor error for the unperturbed trial; (c) The A/P CG velocity attractor during an perturbed cadence trial; (b) The attractor error for the perturbed trial. Note the delay response in the attractor relative to the cadence change (it will require at minimum one step to realize the beat has changed).

Figure 3 illustrates for a representative HE participant the two dimensional attractor and reference trajectory for A/P velocity of the CG during a repeated stepping test with no cadence perturbation (Figure 3a and 3b), and with a cadence perturbation (Figure 3c and 3d). The calculated error for the attractors (left panel) are shown in error plots (right panel). The sharp transition in the error at 11–12 seconds (Figure 3d) corresponds to the cadence transition from 100 steps/minute to 80 steps/minute. Figure 3d indicates that the HE participant was able to return to a stable trajectory within 2 to 3 cycles, though the error remained slightly higher than prior to the perturbation.

**Figure 4:**
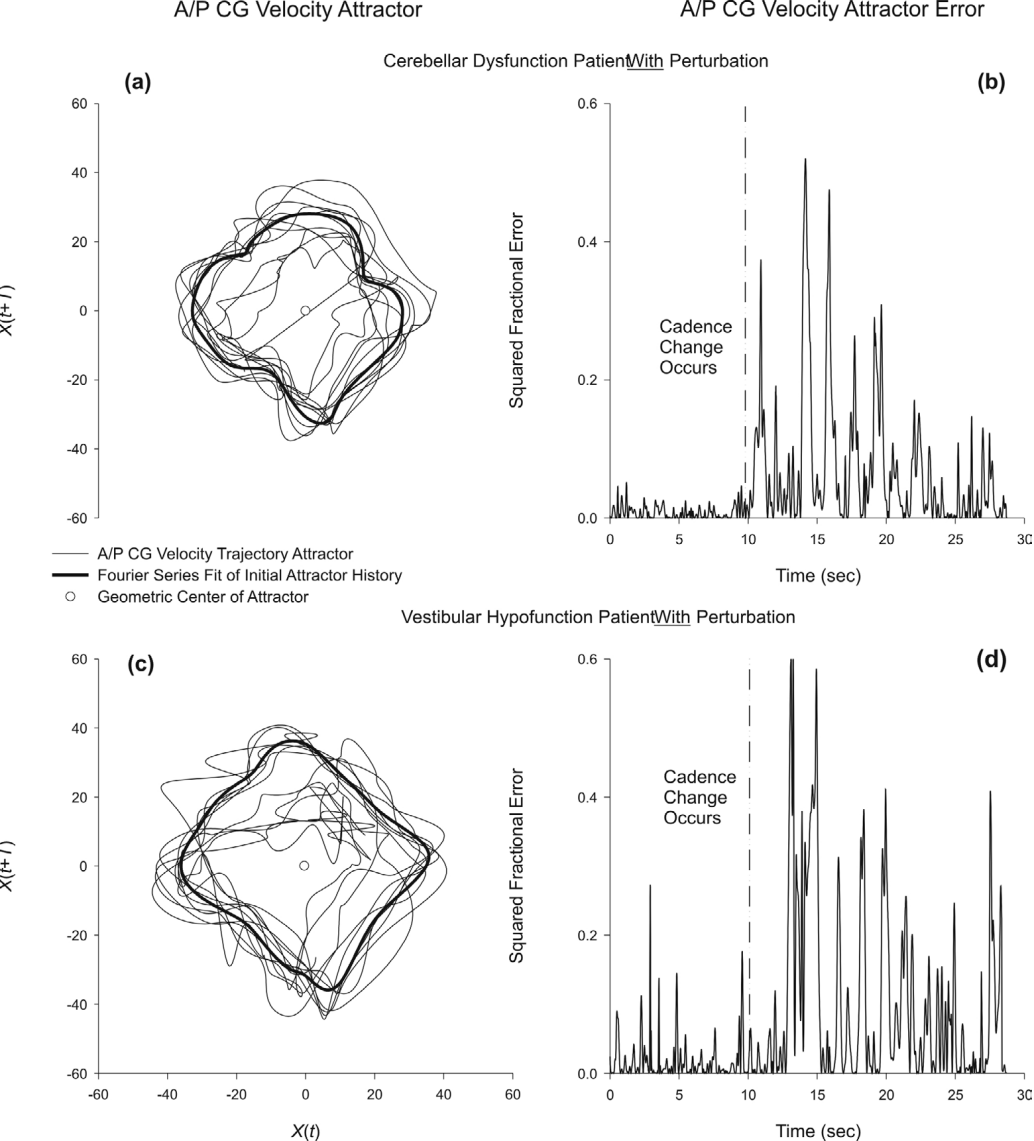
Attractor trajectories for two representative balance impaired patients  during the stepping task. The top panels are for a patient with cerebellar  dysfunction: (a) The A/P CG velocity attractor during a perturbed cadence  trial; (b) The attractor error for the perturbed trial. The bottom panels  are for a patient with vestibular hypofunction: (c) The A/P CG velocity  attractor during a perturbed cadence trial; (b) The attractor error for the  perturbed trial. As with the healthy subject (see Figure 3), there is a time  delay between perturbation onset and response of the attractor, however,  this particular cerebellar subject was suddenly confused by the change and  momentarily lost the pace.

Representative stepping perturbation data for a VH and a CB participant are shown in Figure 4. The left panels of Figure 4 demonstrate erratic attractor behavior in these individuals, and the right panels of Figure 4 shows the resulting error calculations for these participants. Compared to the HE subject in Figure 3, data in Figure 4 shows that a return to a stable trajectory does not occur within 2 to 3 cycles for those with balance disorders. As with the healthy subject (see Figure 3d), there is a time delay between perturbation onset and response of the attractor. Error measures (Emax, Emin and Edif) for all participants are summarized in Table 2.

**Figure 5:**
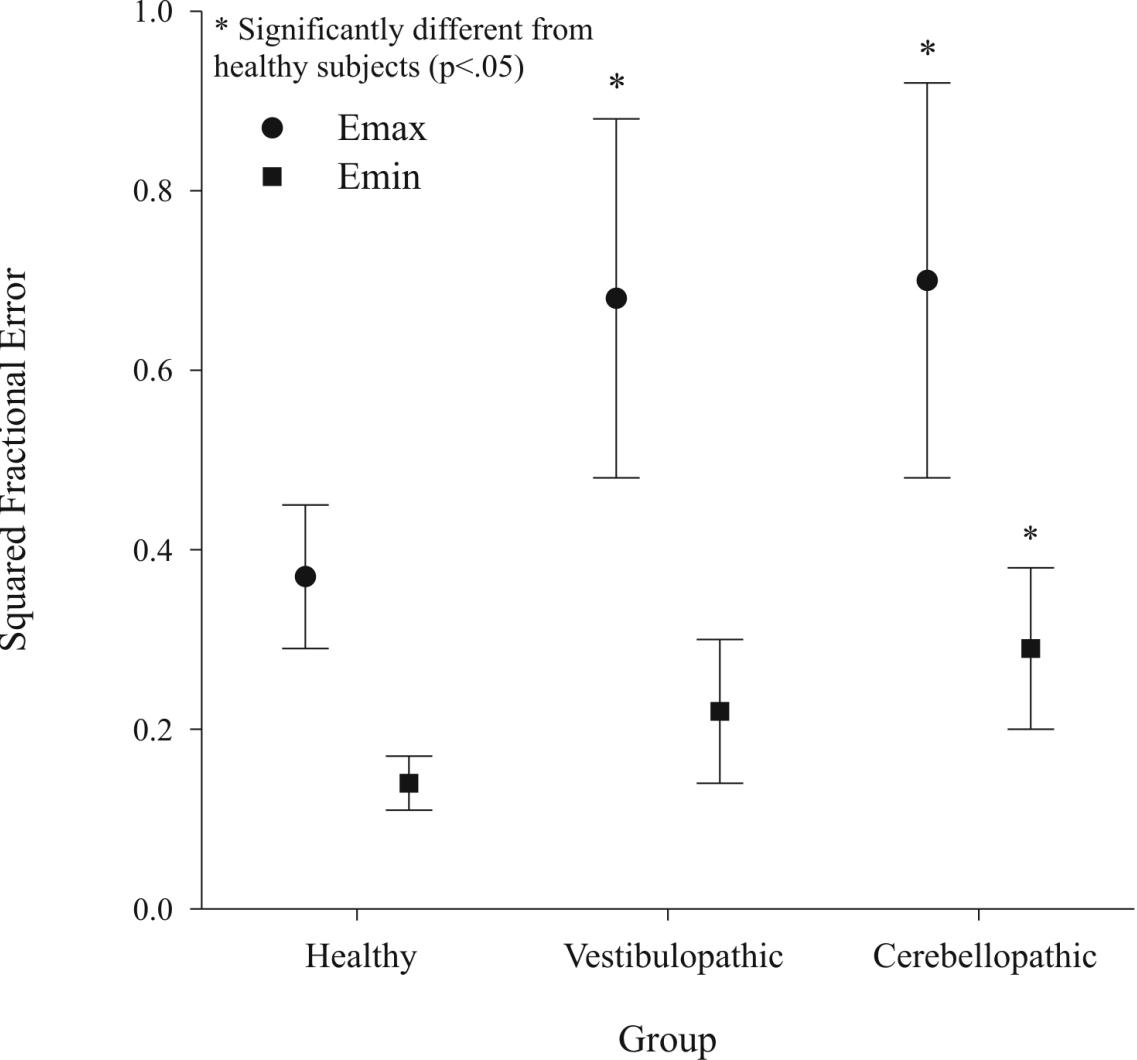
Maximum (Emax) and minimum (Emin) peak squared fractional errors from 2 second interval bins during 10 seconds following the perturbation. Peak error Emax at perturbation (*p *= .019) and peak error Emin after 10 seconds (*p *= .028) were greater for balance impaired patients compared to healthy subjects.

Our hypothesis that balance impaired participants would demonstrate a greater perturbation response than healthy participants, as measured by the fractional error variables, was supported. One-way ANOVA revealed significant between-groups differences for Emax (*p *= .019), and Emin (*p *= .028). Both balance impaired groups had significantly higher Emax than HE participants (CB: *p *= .049; VH: *p *= .026), but were not different from each other (*p *= .985). Using age and weight as covariates did not change the significant outcomes; both Emax and Edif were significantly different between groups (*p *= .027 and *p *= .023, respectively) when controlling for these potentially confounding variables. Mean errors for the CB group, VH group and the HE group are shown in Figure 5. It should be noted that the highest error observed (.94) was for both a CB and a VH participant (Table 2).

Our hypothesis that balance impaired participants demonstrate a slower recovery to the perturbation response than healthy participants was also supported. Although Emin was significantly different between groups (*p *= .028), it was only significantly higher for CB participants compared to HE participants (*p *= .026); there was no significant difference between VH and HE participants (*p *= .147). Interestingly, the between-groups differences in Edif approached the level of significance (*p *= .051). The reason became clear when Edif was expressed as percent decease: all three groups decreased their error by approximately 60% in the 10 second interval following onset of the cadence perturbation: the recovery time for balance impaired participants was longer than for healthy participants because their error response was so much higher.

## Discussion

While measures of standing stability are commonplace, measures of locomotor stability in balance impaired individuals are few [29,31-34,39]. In this report we describe a locomotor perturbation test and analytical procedure for quantifying postural control during a dynamic functional motor task.

The findings of the present study indicate that both balance impaired groups (vestibular hypofunction and cerebellar pathology) revealed a more variable stepping pattern and a slower recovery as a result of the cadence perturbation compared to the healthy participants, suggesting the balance impaired individuals experienced difficulty maintaining fluid movement during the trial, with a diminished ability to predict future position of the whole body CG. However, as shown by Table 2 and Figure 5, our data do not discriminate between peripheral and central vestibulopathy, or within a diagnostic group (bilateral vs. unilateral vestibular hypofunction); indeed, a larger study would be needed to test the power of the protocol and analytical method for this purpose.

While the error means for both balance impaired groups were not statistically different, and the highest error response (.94) was observed in both the CB and VH participants, the most interesting responses were observed in the CB group. Although qualitative, observation of computer animated stepping trials suggested that two of the three CB participants were unable to smoothly adjust their stepping cadence when the cadence perturbation was applied, and appeared to have difficulty regaining the inter-limb coordination required to match the new metronome beat. This supports our previous finding that people with CB have poor inter-limb coordination during a repeated stepping task compared to their healthy counterparts [32]. Furthermore, Timman and Horak [40] found that participants with cerebellar pathology are less able to scale anticipatory postural adjustments when stepping was cued with a backward translation of the support surface. Our data suggests that cerebellar pathology also affects the ability to scale postural adjustments during unanticipated cadence perturbation.

VH participants had a slightly, though not significantly, lower error response than CB participants, and had significantly higher error response compared to HE participants. This latter finding also supports our previous reports that people with VH are less stable [34] and less smooth [33] during a stepping task than are their healthy counterparts. The perturbation response for the VH group was probably not due to difficulty controlling interlimb coordination, but rather, due to cadence corrective action (after the perturbation) coming too late to slow down the center of gravity after the perturbation is cognitively realized. The late corrective action, allowing the attractor trajectory to deviate further from its orbit, was perhaps due to additional time required of visual and proprioceptive mechanisms to re-assert control over head and gaze stability.

Van Emmerik and Wagenaar [29] studied the relative phase and frequency dynamics of interlimb coordination and trunk rotation during walking in people with Parkinson's disease (PD) and healthy participants when systematically varying walking speed. Their findings revealed that people with PD often have a reduced ability to switch between walking patterns and relatively more stable coordination patterns compared to young healthy participants. They hypothesized that the hyper-stable coordination patterns in PD cause a reduced flexibility (that is, ability to switch between coordination patterns). The results of the present study indicate that the balance impaired individuals have a larger variability in stepping behavior and a slower recovery (longer relaxation time) as a result of the perturbation. It suggests that a hypo-stable stepping pattern results in a slower recovery from a perturbation, which makes, for example, balance impaired individuals more at risk for falls.

Van Wegen et al. [28] reported that healthy elderly and people with PD show a decreased time-to-contact variability in body sway during quiet standing in the medio-lateral direction; older adults and people with PD remained a larger distance from their stability boundary than young participants. In addition, it was found that during walking, in the higher frequency ranges (3–12 Hz), younger participants had higher power than the older participants, while in the lower frequency ranges (0–3 Hz), the older participants had higher power than the younger participants (see also van Emmerik et al. ..[30]). In their approach to coordination, fluctuations i.e., variability, can play a functional role in the stabilization and adaptation of coordination patterns. From this perspective, a reduction in variability (hyper-stability) also has a negative impact on movement coordination. The findings of the present study strongly suggest that in people with peripheral and central vestibulopathy the flexibility of movement coordination is reduced (increased variability) as a result of hypo-stable stepping patterns. On the basis of the above-mentioned findings we hypothesize that a similar problem in stability and flexibility during stepping or walking may exist in healthy elderly at risk for falls.

The shape of the attractor in Figure 3 for a HE subject resembles a diamond and has closely packed orbital trajectories. When a cadence perturbation is applied, the predictive quality of the attractor breaks down during the transition from a 100 bpm orbital trajectory to an 80 bpm orbital trajectory. Even the HE subject shown in Figure 4 required two to three steps to restabilize the new trajectory. Participants with peripheral (VH) and central (CB) vestibulopathy disorders did not transition as smoothly as HE participants when moving between 100 bpm and 80 bpm, however, they appeared to adapt at a similar rate over a 10 second interval. Testing for a longer interval at 80 bpm following the perturbation onset, however, would be required to determine if indeed there are differences in recovery rate; the need for longer testing was exemplified by the fact that error magnitudes did not return to pre-perturbation levels for any participants.

It is important to note that the recovery time following perturbation depends on when the perturbation occurs within the stepping cycle, and the feedforward nature of volitional stepping. These factors probably contribute to the variability in Emax and Emin times, and hence influence the recovery time response, more so than system time constants (such as the 6 msec VOR response or 100 msec "long loop" response to the brain and back to muscle [41]).

The cadence, and cadence transition, applied for participants may also be a factor influencing the results. To assess the sensitivity of the attractor geometry to the stepping rate, and thereby provide a rationale for the cadence perturbation rates chosen to conduct on participants, we examined the attractor geometry for several participants (not included in this study) who performed the stepping trials at different cadences (60–152 bpm). When plotting the attractor radius against stepping rate (data not presented here), we found a curvilinear relationship suggesting the attractor radius peaks in dimension at about 120 bpm, but that the difference between 100 bpm and 80 bpm was sufficiently large and linear. We concluded that our choice of a cadence perturbation was appropriate for the participants studied.

It is important to note that we did not quantify hearing ability of the study participants, although no participant indicated hearing impairment on their entry medical screening. Quantifying hearing ability would be important for a larger study because the perturbation requires one to detect the metronome transition. Because the sample was small, we also chose to ignore the gender of the participants. Indeed, a larger study would not ignore such influences. Furthermore, there were differences in age (though not statistically significant) and weight (significant at *p *= .05 between VH and HE) among groups that cannot be ignored, as response latency in concurrent cognitive tasks may be influenced by age-related and other impairment [42]. However, because we found that the between-groups differences persisted when age and weight were used as covariates, we are confident in our conclusion that balance-impairment explained the majority of the differences observed between groups. We also analyzed only the velocity perturbations in the anterior-posterior direction. It is reasonable to expect that a similar analysis of the medio-lateral velocities may yield interesting results.

In cases where the body CG cannot be estimated accurately (for example, when using systems that only track a few body segments), other measures, such as pelvis or trunk marker velocities should be compared to the results in this analysis which uses the whole body CG velocity derived from an 11 segment inertial body model [38,43,44].

We conclude that the cadence perturbation test is useful for quantifying locomotor stability control in people with peripheral or central vestibulopathy. People with damaged vestibular systems or those with cerebellar damage performed significantly worse on the cadence perturbation tests compared to healthy participants. Clearly our results are not necessarily generalizable due to the small pilot study sample used and the above identified limitations; however, the data presented do suggest that the tool used to quantify stepping stability, derived from non-linear dynamics, is useful and sensitive enough to detect the effects of stepping cadence changes when controlled by external auditory cues.

## Competing interests

The author(s) declare that they have no competing interests.

## Authors' Contributions

All authors participated in the overall study design, contributed to the interpretation of data and writing/editing of the manuscript, and have read and approved the final manuscript. CAM conceived the hypotheses, developed and programmed the non-linear dynamic analysis methods, carried out the data analysis, and prepared the manuscript; DEK was the principal investigator of the project; RW was the project consultant.
